# Simultaneous Estimation of Rebar Diameter and Cover Thickness by a GPR-EMI Dual Sensor

**DOI:** 10.3390/s18092969

**Published:** 2018-09-06

**Authors:** Feng Zhou, Zhongchang Chen, Hai Liu, Jie Cui, Billie F. Spencer, Guangyou Fang

**Affiliations:** 1 School of Mechanical Engineering and Electronic Information, China University of Geosciences (Wuhan), Wuhan 430074, China; zhoufeng@cug.edu.cn (F.Z.); chenzhongchang@cug.edu.cn (Z.C.); 2 School of Civil Engineering, Guangzhou University, Guangzhou 510006, China; jcui@gzhu.edu.cn; 3 Department of Civil and Environmental Engineering, University of Illinois at Urbana-Champaign, Urbana, IL 61801, USA; bfs@illinois.edu; 4 Institute of Electronics, Chinese Academy of Sciences, Beijing 100190, China; gyfang@mail.ie.ac.cn

**Keywords:** non-destructive testing (NDT), ground-penetrating radar (GPR), electromagnetic induction (EMI), rebar detection

## Abstract

Precise characterization of reinforcing bars (rebars) in a concrete structure is of significant importance for construction quality control and post-disaster safety evaluation. This paper integrates ground-penetrating radar (GPR) and electromagnetic induction (EMI) methods for simultaneous estimation of rebar diameter and cover thickness. A prototype of GPR-EMI dual sensor is developed, and a calibration experiment is conducted to collect a standard EMI dataset corresponding to various rebar diameters and cover thicknesses. The handheld testing cart can synchronously collect both GPR and EMI data when moving on the concrete surface, from which a data processing algorithm is proposed to simultaneously estimate the rebar diameter and cover thickness. Firstly, by extracting the apex of the hyperbolic reflection from the rebar in the preprocessed GPR profile, the rebar position is determined and further used to extract the effective EMI curve. Then, the rebar diameter and cover thickness are simultaneously estimated from the minimum mean square error between the measured and calibrated EMI data under the constraint of the GPR-estimated cover thickness. A laboratory experiment is performed using four casted concrete specimens with 11 embedded steel rebars. The results show that the diameters of 10 rebars are correctly estimated out of the 11 rebars, and the maximum estimation error for the cover thickness is 6.7%. A field trial is carried out in a newly-constructed building, and the diameters of four tested rebars are all accurately estimated while the estimation errors of the cover thickness are less than 5%. It is concluded that the developed GPR-EMI dual sensor and the proposed algorithm can estimate the rebar diameter and cover thickness accurately by a single scan.

## 1. Introduction

Non-destructive testing (NDT) techniques play a significant role in monitoring and diagnosing construction structures. Accurate characterization of the properties of reinforcing bars (rebars) in concrete structures is critical for the quality control during the construction phase, as well as for health monitoring and post-disaster safety evaluation during the operation phase [1]. The parameters of rebars that need to be inspected include their location, spacing, diameter, cover thickness and the degree of corrosion. Among them, accurate determination of the rebar diameter and cover thickness in a non-destructive way is still challenging [2], which is the objective of this paper.

Electromagnetic induction (EMI) is the principle of most of the commercially available rebar locators and cover meters [3]. An EMI sensor consists of magnetic coils, which excite time-varying magnetic fields towards the concrete and receive the induced secondary magnetic fields from conductive objects [4,5]. When an EMI sensor is used for rebar inspection, the induced secondary magnetic fields are sensitive to both the rebar diameter and cover thickness. Thus, a rebar locator or cover meter can estimate the rebar diameter or cover thickness, only if the other one is known, with the pre-calibrated data of the EMI strength stored in the instrument memory [6,7]. A curvilinear model was developed to estimate the rebar diameter and cover thickness through the peak amplitude and the full width at half height (FWHH) extracted from the measured EMI pulse response, and the results show that the accuracy of the estimated cover thickness is much higher when the rebar diameter is given than that when the rebar diameter is unknown [8]. A laboratory experiment was carried out to assess the capability of the commercial EMI instruments in estimating rebar diameter and cover thickness, and the results show that estimation errors rise with the increase of cover thickness and the instruments become unreliable [9]. By acquiring two EMI readings at different measurement heights or employing two vertically-spaced coils, it is possible to simultaneously estimate the rebar diameter and cover thickness, but it is often inconvenient to scan in congested metal work areas and difficult to avoid the mutual interference of two sets of coils [10]. Neural networks were trained to estimate rebar diameter and cover thickness respectively, and the estimation accuracy satisfies the industrial standards [11]. Ultrasonic echo was combined with EMI measurement for mapping the meshed reinforced concrete and estimating the cover thickness, where better results were obtained than those from an alone EMI survey [12]. However, this method requires to know rebar diameters in advance, and it cannot solve the rebar size.

Ground-penetrating radar (GPR) is another important NDT method based on the propagation and scattering of high-frequency electromagnetic (EM) waves. It has been successfully applied to utility detection [13], pavement inspection [14,15], environmental studies [16], oil monitoring [17], space exploration [18], etc. Due to the great contrast of electrical properties between the steel rebars and the concrete background, rebar is a favorite target for GPR detection [19]. Recently, increasing interests have been paid to the determination of the geometric properties of rebars (e.g., diameter, spacing and buried depth) [20,21,22], the moisture of concrete [23,24] and the degree of rebar corrosion [25,26]. In a GPR profile with the survey line orthogonal to the rebar direction, the reflection from a rebar can be approximated as a hyperbola. Cover thickness can be estimated by the hyperbolic apex once the EM velocity in the concrete is accurately estimated [22]. However, the EM velocity in concrete can hardly be accurately estimated by experience or a simple GPR measurement, since concrete is heterogeneous and its dielectric properties are dependent on the texture of the mixture [27,28]. A conic equation relating the rebar diameter, cover thickness and EM velocity in concrete was developed [29], and fitting between the extracted trajectory of the rebar reflection and the modelled hyperbolic curve was used to estimate the rebar diameter, cover thickness and wave velocity [30,31,32]. However, studies find that the shape of the hyperbolic curve is insensitive to the rebar diameter, therefore it is not easy to straightforwardly infer the rebar diameter through the hyperbolic fitting [22]. To avoid directly picking up or extracting the trajectory of the rebar reflection, Hough transform and its enhanced version were applied to estimate the diameter of a buried cylindrical object [33]. An empirical procedure was presented to estimate rebar diameters by associating the antenna footprint with the power reflectivity from the rebar [20]. However, the estimation accuracy may suffer from the instability of an impulse GPR system [34]. Through a multi-polarization GPR measurement, the rebar diameter was estimated through the ratio of the reflection amplitudes recorded in different polarization channels. However, the authors acknowledge that the method is sensitive to the wavelength of the GPR employed and that the estimation accuracy depends on an optimal selection of the GPR frequency versus the rebar diameters [21].

EMI is sensitive to both the depth and size of metallic buried objects. However, it is difficult to simultaneously and accurately obtain the two unknowns in a direct manner. GPR has a high sensitivity on the buried depth rather than the size of the objects, whereby a straightforward estimation of the buried depth is readily done by a time-depth conversion [22]. Considering the respective advantages of EMI and GPR, an associated survey or designed system integrating EMI and GPR has been applied to landmine detection [35], pollution evaluation [36,37] and soil moisture prediction [38]. This paper proposes to integrate EMI and GPR for simultaneous and accurate estimation of rebar diameter and cover thickness. Operating the separate EMI and GPR devices for synchronous data collection is feasible, but it has some disadvantages, such as low efficiency, location deviation and complicated data fusion [39]. For that reason, we develop a compact and handheld prototype integrating EMI and GPR for convenient operations and fast measurements [40], and a method for simultaneous estimation of rebar diameter and cover thickness is proposed.

## 2. GPR-EMI System

### 2.1. System Description

**Figure 1:**
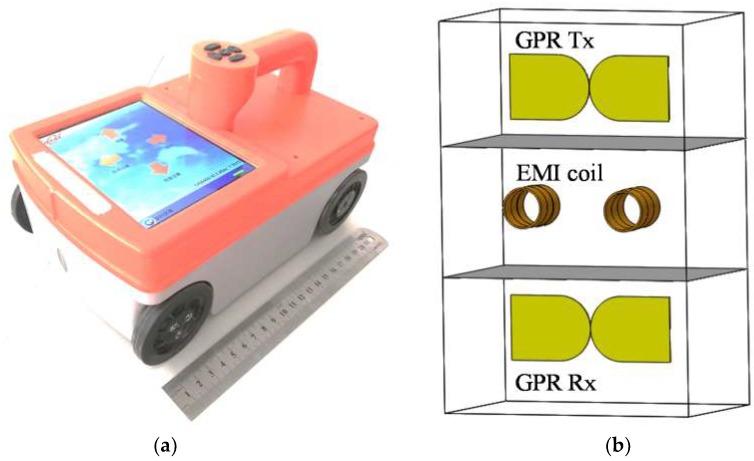
(**a**) Photo of the prototype of the developed rebar detection system, and (**b**) schematic structure of GPR antennas and EMI coils inside the apparatus.

In order to carry out a synchronous measurement of both EMI and GPR data, a novel dual-sensor system has been developed [40]. Figure 1a,b shows a photo of the developed prototype and its schematic structure, respectively. This compact and handheld testing tool consists of a GPR module, an EMI module, control unit and display, facilitating in-situ rebar scanning. The GPR module employs a pair of antennas for transmitting and receiving electromagnetic waves, respectively. The antennas have an improved bowtie shape with a center frequency of 1.6 GHz. The effective bandwidth ranges from 0.9–2.5 GHz. The EMI module employs a pair of magnetic coils. One coil is used for transmitting magnetic fields into concrete, and the other for receiving secondary magnetic fields generated from the eddy currents on the embedded metallic rebar. Each coil has 120 turns with a diameter of 3 cm, and the working frequency is 40 KHz. The system has a total power of 7 watts. Four wheels are installed at the bottom of the device body enabling a convenient movement on the concrete, and one of them is equipped with a distance-measuring encoder, which is used to trigger the transmitters. In addition, an LCD display is installed at the top of the device to show EMI and GPR data in real time.

**Figure 2:**
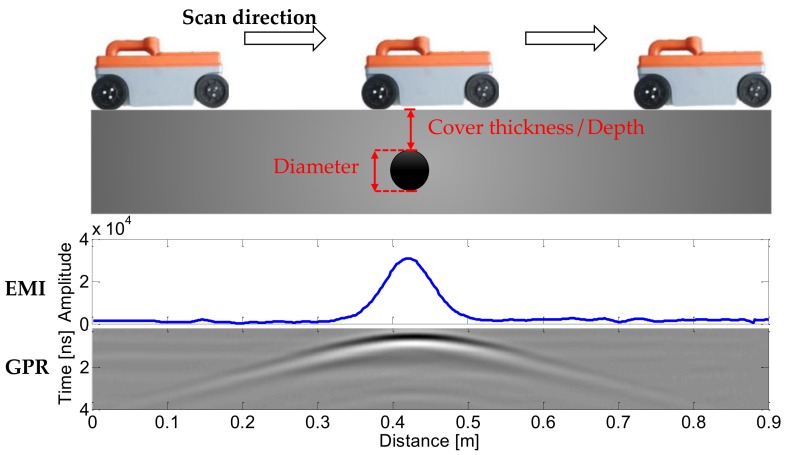
Schematic illustration of the measurement process using the developed GPR-EMI dual sensor and an example of measured EMI and GPR data.

While operating, the device is placed on the concrete surface, and moves across the potential rebars. Radar and EMI sensors implement synchronous scanning, and both sets of data are displayed on the display in a real-time pattern. Figure 2 illustrates the operation process and data display. The EMI response on the embedded rebar is a pulse-shape curve, while the radar response is a hyperbolic reflection, of which the apexes are right above the rebar.

### 2.2. EMI Calibration

**Figure 3:**
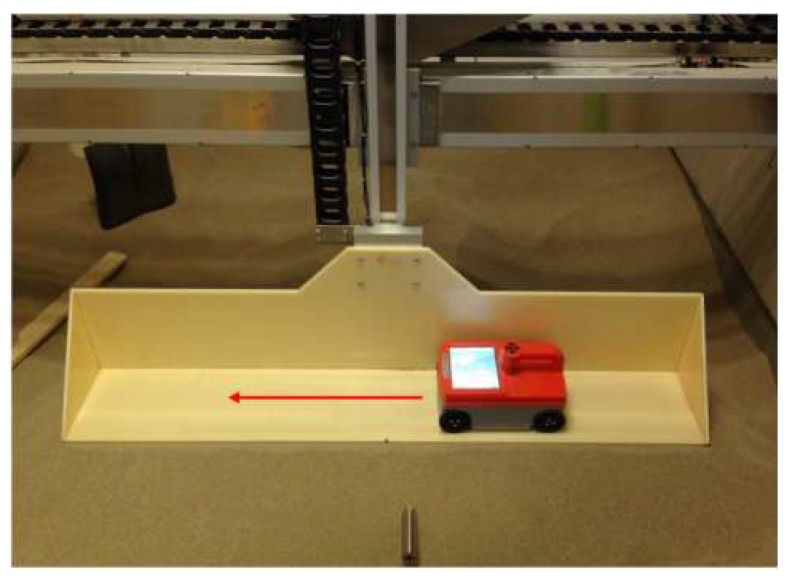
The experimental platform for EMI calibration.

The calibration of rebar data by EMI sensing is carried out using a customized Cartesian-coordinate scanning frame installed above a sandpit as shown in Figure 3. The sandpit is filled with dry sand, which has a dielectric permittivity of about 3, and rebars of varying diameter are buried in the sand at different depths. We have verified through additional measurements (not shown here) that the EMI sensor has almost the same rebar responses against a background of air, dry sand and concrete materials. The primary reason is that these types of background materials have extremely low conductivity and magnetic permeability compared with the buried steel rebars [32]. Thus, the dielectric properties of a low-loss dielectric background medium have little influence on the EMI response. A 5-mm thick plastic platform is installed on the scanning system, enabling an automated movement in the vertical and horizontal directions. The GPR-EMI dual sensor moves on the plastic platform to avoid the wheel slipping and data missing. The plastic platform can also be used to wipe away the top sand, ensuring a flat sand surface and an accurate control of the rebar cover thickness.

In our calibration experiments, 11 steel rebars are chosen as the calibration samples referring to the industrial construction standard in China [41]. The rebar diameters are respectively 6, 8, 10, 12, 14, 16, 18, 20, 22, 25 and 28 mm. The rebars are sequentially buried in the dry sand at gradually decreased depths from 60 mm to 5 mm in steps of 1 mm. For each cover thickness and diameter of the rebars, we implement a cross-sectional EMI scanning on the platform.

**Figure 4:**
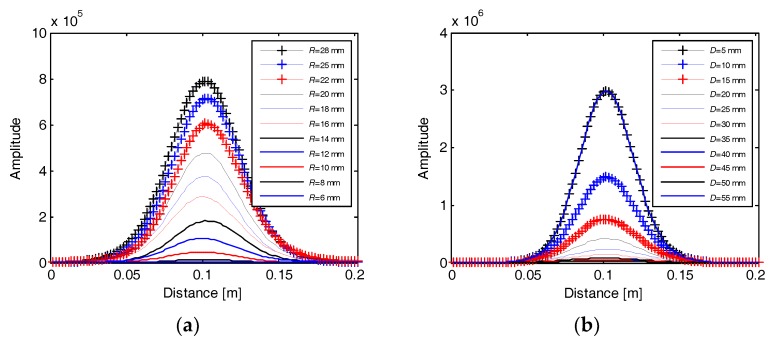
Recoded EMI curves in the calibration experiment when (**a**) *D* = 20 mm and (**b**) *R* = 20 mm. *D* and *R* are the cover thickness and diameter of the rebars, respectively.

**Figure 5:**
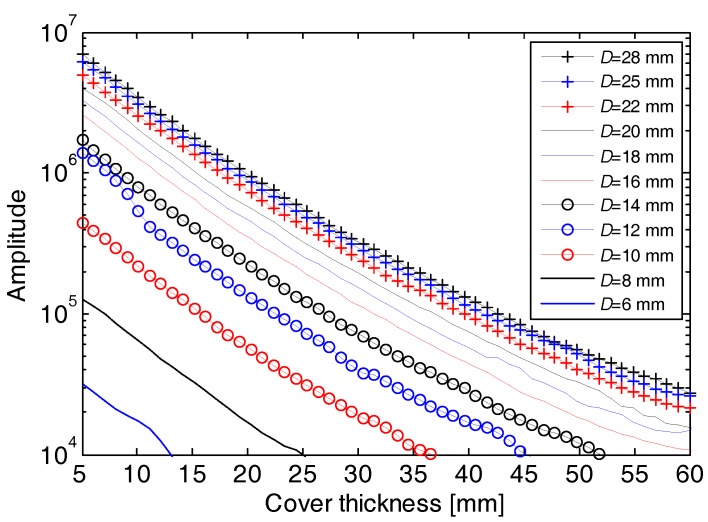
Variation of the EMI peak amplitudes versus the cover thickness and rebar diameter when the coils are right above the rebars during the calibration experiment.

The calibration process records 616 EMI response curves corresponding to the eleven rebar diameters and 56 cover thicknesses. Figure 4a,b shows two sets of EMI curves responding to a fixed cover thickness (*D* = 20 mm) with varying rebar diameters and a fixed rebar diameter (*R* = 20 mm) with varying cover thicknesses, respectively. It can be seen that all the EMI curves exhibit a pulse response on the buried rebars. The signal strength raises proportionally with the increase of the rebar diameter, while drops exponentially with the increase of the cover thickness, reflecting a high sensitivity of EMI signal to both the rebar diameter and cover thickness. The amplitude of the peak of each EMI curve is picked up, and its variations with the rebar diameters and cover thicknesses are shown in Figure 5. The high sensitivity of the peak amplitude to both the rebar diameter and cover thickness indicates that it is able to estimate the rebar diameter once the cover thickness is known, and vice versa. However, we can see that a peak amplitude can correspond to different pairs of rebar diameter and cover thickness, which means a large error may exist when estimating both rebar diameter and cover thickness from the peak amplitude with no prior information. Therefore, we propose to integrate the GPR and EMI data for simultaneous estimation of the rebar diameter and cover thickness. A data processing algorithm is presented in the next section.

## 3. Data Processing

**Figure 6:**
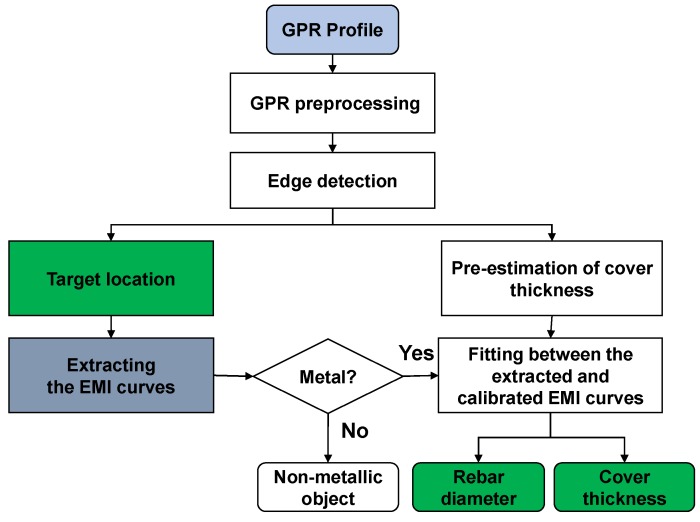
Flow chart of the proposed algorithm for simultaneous estimation of rebar diameter and cover thickness from the recorded GPR and EMI data.

The data processing algorithm for simultaneous estimation of rebar diameter and cover thickness from the GPR and EMI data is depicted in Figure 6.

**Figure 7:**
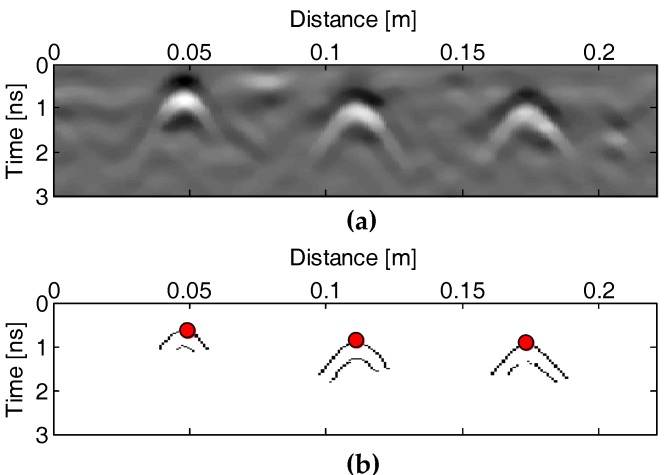
(**a**) GPR profile after preprocessing and (**b**) the corresponding binary image after the edge detection.

The detailed information is described as follows:

(a) The GPR data is preprocessed to increase the signal to noise ratio. A sequence of standard GPR processing techniques are implemented, including DC removal, zero-time correction, band-pass filtering, amplitude scaling, median filtering, and background removal [42].
(b) The hyperbolae from the buried rebars in the GPR profiles are extracted by an edge detection algorithm after pre-processing, and their apex coordinates are picked up for locating the rebar and roughly estimating the cover thickness, as shown in Figure 7. The Sobel operator is used for the edge detection [43]. Figure 7a,b respectively show the preprocessed GPR profile and the binary image after the edge detection. It shows that the Sobel operator is effective for the rebar hyperbola extraction in the GPR profile even if the signal to clutter ratio is high.

The upper and lower two arc trajectories correspond to the two troughs in the GPR reflection waveforms, and the apex of the upper one is used for the rebar localization.

(c) Localization of the buried rebar and extraction of the effective EMI curve. From the horizontal coordinate of the detected hyperbolic apexes, the horizontal location of buried rebar can be accurately determined, which is further used to extract the effective EMI curves from the embedded rebar. The effective EMI curve centers on the rebar location with the span defined as twice of the FWHH, and it contains the majority of the useful information in the EMI response while stays away from the noise level. The EMI amplitude, with the location corresponding to the hyperbolic apex of the GPR profile, is used to judge whether the GPR hyperbola is reflected by a rebar or a plastic pipe. If the EMI amplitude is close to the noise level, the reflective object is judged as non-metallic; otherwise, it is judged as a metallic rebar.
(d) Pre-estimation of the cover thickness. Knowing the time coordinate (*t*) of the hyperbolic apex, the two-way travel time of EM waves propagating from the air-concrete interface to the embedded rebar is used to roughly estimate the cover thickness by:
  (1) $$D=\frac{1}{2}vt,$$
  where *D* is the cover thickness (m), *v* is the EM wave velocity in the concrete (m/ns), and *t* is the two-way travel time (ns). The velocity is calculated by:
  (2) $$v=\frac{c}{\sqrt{\varepsilon_{r}}},$$
  where *c* is the EM wave velocity in free space, i.e., 0.3 m/ns, and *ε*_r_ is the relative permittivity of the concrete. Taking the heterogeneity of the concrete compositions and the intrusive moisture into account, we assign *ε*_r_ ranging from 4 to 10 [44,45]. The permittivity is utilized to estimate the possible range of rebar cover thickness, which is used as a constraint condition for the accurate estimation of the rebar diameter and cover thickness in the following step.
(e) Determination of the rebar diameter and cover thickness. Through calculating the mean square errors between the EMI data extracted from the field measurement and those calibrated in the laboratory in advance (as stated in the previous section), the rebar diameter and cover thickness are simultaneously estimated through searching the local minimum mean square error under the constraint of the GPR-estimated cover thickness. The error function is expressed by:
  (3) $$MSE(i,j)=\frac{1}{l}\int_{-\frac{l}{2}}^{\frac{l}{2}}{\left[f(x)-g_{i,j}(x)\right]}^{2}dx,$$
  where *f*(*x*) is the in-situ measured EMI curve, $g_{i,j}(x)$ is the calibrated EMI curves, *i* and *j* are respectively the serial numbers standing for various rebar diameter and cover thickness in the calibration experiment, *x* is the horizontal coordinate of the EMI curve, *l* is the intercept length of the extracted effective EMI curve, which is twice of the FWHH as stated above, and *MSE* is the mean square error. The search of the local minimum *MSE* is implemented under the constrained scope of the cover thicknesses that are estimated by GPR data in the previous step. This constraint condition avoids multiple solutions of rebar diameter and cover thickness, and thus can improve the estimation accuracy.

## 4. Laboratory Experiments

To check the proposed method for simultaneous estimation of rebar diameter and cover thickness, we conducted a laboratory measurement with the developed GPR-EMI device using four concrete specimens embedded with rebars.

### 4.1. Experimental Setup

**Figure 8:**
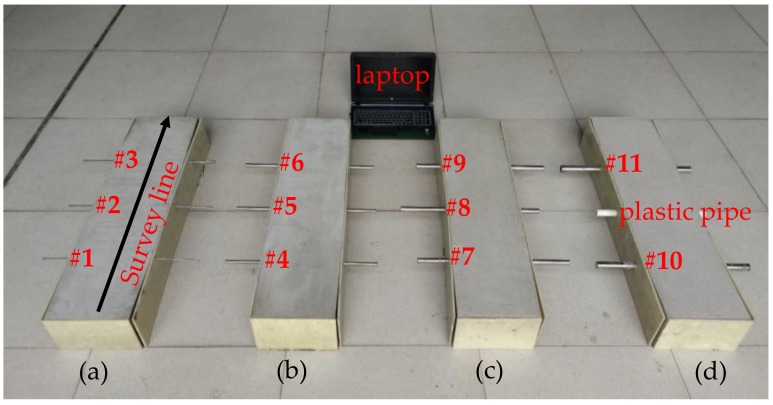
The four cast concrete specimens (**a**–**d**) with 11 steel rebars and one plastic pipe embedded inside.

In the laboratory experiment, four concrete specimens were casted and eleven rebars with different diameters were embedded inside. The specimens were poured with the ordinary Portland concrete, and the dimensions are 1000 mm × 250 mm × 150 mm. The rebars, labelled from #1 to #11 with the diameters ranging from 6 to 28 mm, were buried in the concrete specimens with different cover thicknesses, as shown in Table 1 and Figure 8. A plastic pipe was embedded in the middle of the fourth specimen for a comparison test. Measurements using the developed GPR-EMI dual sensor were conducted on the surface of the specimens along the direction orthogonal to the rebars after one-month curing period in a common environment.

### 4.2. Results

**Figure 9:**
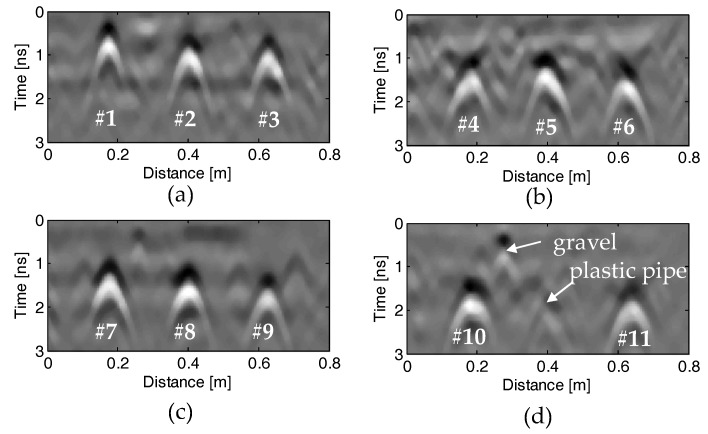
Recorded GPR profiles on the four concrete specimens (**a**–**d**) after pre-processing. The serial numbers of the tested rebars are marked below the hyperbolic reflections, and the reflections from the plastic pipe and gravel are indicated.

**Figure 10:**
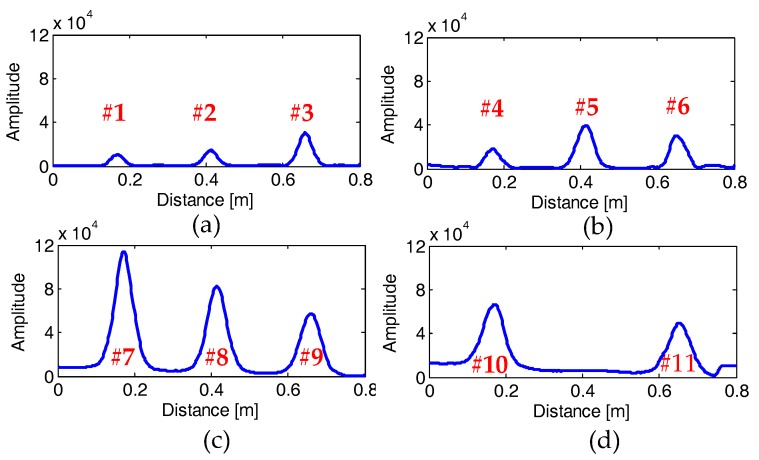
Recorded EMI curves on the four concrete specimens (**a**–**d**) after pre-processing. The serial numbers of the tested rebars are marked above or below the impulse EMI response of the eleven rebars.

Figure 9a–d or Figure 10a–d show the GPR profiles and the corresponding EMI response curves measured on the four specimens, respectively, after data pre-processing. We can see that each rebar is represented as a strong hyperbolic reflection in the GPR profile and an impulse response in the EMI curve, respectively. In contrast, the plastic pipe shows a weak hyperbolic reflection in Figure 9d and no response in Figure 10d. It is obvious that the hyperbolic reflection from the plastic pipe is much weaker than those from its neighboring rebars. However, the multiple reflections from the top and bottom of the pipe may be used to infer the diameter of the plastic pipe [46,47]. Therefore, the proposed algorithm distinguishes this hyperbolic reflection coming from a non-metallic object and does not estimate its diameter and cover thickness. Besides, some hyperbolic reflections from large gravels embedded in the concrete can also be observed in the GPR profiles, as shown in Figure 9d.

**Figure 11:**
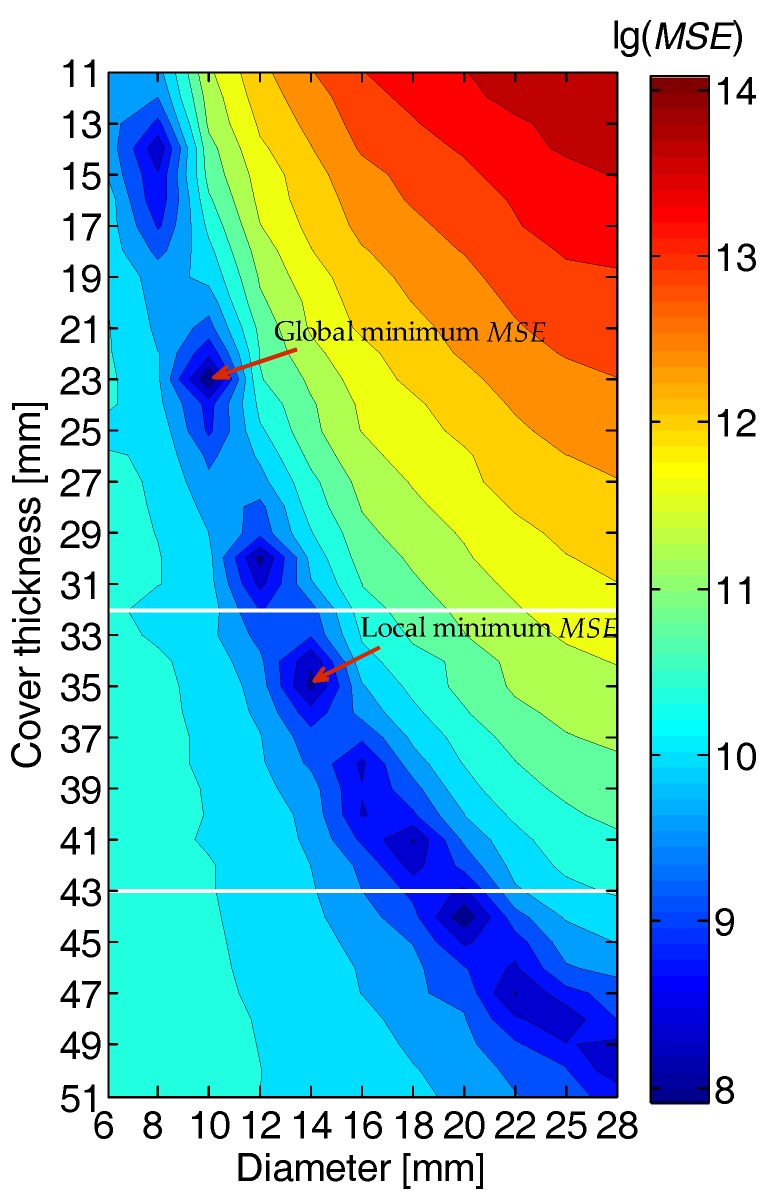
Contour plot presenting the *MSE*s calculated between the measured and calibrated EMI data for simultaneous estimation of the rebar diameter and cover thickness. The range of the GPR-estimated cover thickness is marked by two horizontal white lines. The global minimum *MSE* and local minimum *MSE* are respectively indicated by the red arrows.

From the hyperbolic reflections of the eleven embedded rebars, the rebar locations are determined by the aforementioned edge detection algorithm, and the corresponding effective EMI curves are extracted. For each measured EMI response, we extract the effective EMI curve and calculate a map of *MSE*s between the measured and calibrated EMI curves using Equation (3). Figure 11 shows the *MSE*s obtained from the rebar labelled #5, whose actual diameter and cover thickness are 14 and 35 mm, respectively. From the hyperbolic reflection of the rebar in the GPR profile in Figure 9b, the cover thickness of this rebar is estimated in the range of 32–43 mm. This varying range of GPR-Estimated cover thickness considers the uncertainty of the permittivity caused by the moisture and heterogeneity in the concrete, making it possible to find the right local minimum in the *MSE* map for accurate estimation of the rebar diameter and cover thickness. If taking no account of the cover thickness limits, the rebar diameter and cover thickness are estimated to be 10 mm and 23 mm by the global minimum *MSE*, as marked in Figure 11, indicating a large estimation error. However, with the prior information of the cover thickness obtained from the GPR data, the rebar diameter and cover thickness are estimated to be 14 mm and 35 mm, respectively, by the local minimum *MSE*, as marked in Figure 11, which are the same as the true values. This example proves that by combining GPR and EMI data, the estimation accuracy of rebar diameter and cover thickness can be greatly improved.

We estimated the diameter and cover thickness of the eleven rebars in the four specimens, and analyzed the errors between the estimated and true values, as shown in Table 1. Among the eleven rebars, only one has an inaccurate diameter estimation with an absolute error of 2 mm and a relative error of 9.1%. The estimated cover thicknesses present relative errors below 7%. With the increasing cover thickness, there is no evident drop of estimation accuracy observed in the error statistics. Even so, we can observe an exponential decline of EMI amplitudes with the increase of the cover thickness from Figure 4b and Figure 5. This implies that an extraordinarily great buried depth of a rebar is likely to incur an inaccurate or even ineffective estimation because of the low signal-to-noise ratio, as discussed by [9]. The results of the laboratory experiment demonstrate that the developed system and proposed method can estimate the rebar diameter and cover thickness simultaneously and accurately.

## 5. Field Test

### 5.1. Site Description

**Figure 12:**
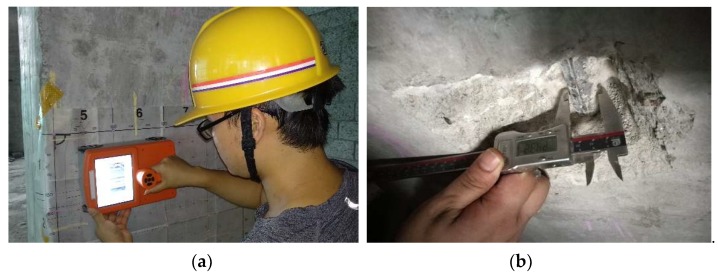
Photos of (**a**) the field operation and (**b**) the drilling measurement.

To further verify the effectiveness of our device in testing practical concrete structures, we chose two reinforced concrete columns in a newly-completed building to conduct a field trial. The columns have a cross-section of 0.8 m × 0.8 m. A quick-scan was implemented to locate the horizontal and vertical rebars. To mitigate strong interference from the neighboring rebars, we set a horizontal survey line between two horizontal rebars, of which the spacing is 15 cm. Figure 12a shows the operation of the GPR-EMI dual sensor in the field. After the EMI and GPR data were collected, the concrete was drilled and the true diameter and cover thickness of the main rebars were precisely measured by a caliper, as shown in Figure 12b.

### 5.2. Results

**Figure 13:**
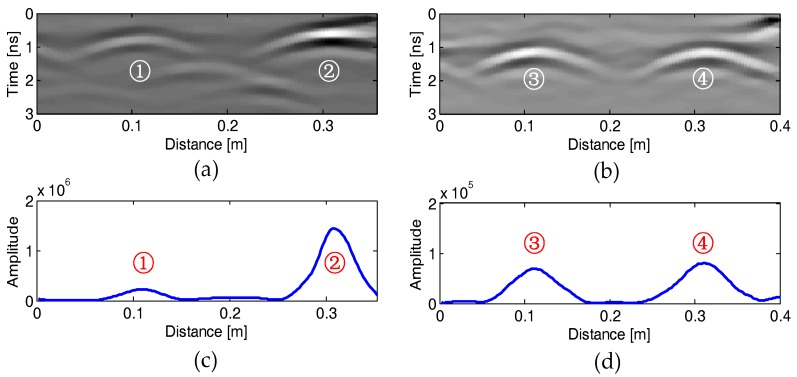
GPR profiles (upper) and EMI curves (lower) recorded over the first (**a**,**c**) and the second (**b**,**d**) columns in the field site. The serial numbers of the detected rebars are marked.

Figure 13a–d shows the recorded GPR profiles and EMI curves on the two columns in the field site. Two rebars were detected for each survey line. The recorded data are used to estimate the diameter and cover thickness of the rebars with our proposed method, and the estimated results are compared with those of the drilling measurement.

Table 2 shows the results of the field test. The results indicate that the rebar diameters are all accurately estimated, and the cover thicknesses have a maximum error less than 5%, reflecting the developed GPR-EMI dual sensor and proposed estimation method are effective for field rebar detection and characterization.

## 6. Conclusions

In this paper, we propose the integration of EMI and GPR for simultaneous estimation of rebar diameter and cover thickness, which is of significance for quality control and safety evaluation of concrete structures. A prototype of GPR-EMI dual sensor has been developed, and a standard set of EMI data for calibration has been recorded using eleven rebars of different diameters buried at different depths in sand. The developed rebar detection device can synchronously record a GPR profile and an EMI response curve by a single scan in a handheld moving manner. The main contribution of this paper is the development of a data processing method for simultaneous estimation of rebar diameter and cover thickness. From the GPR data, a buried object is located and its cover thickness is roughly estimated from the apex of the hyperbolic reflection. The corresponding EMI data is extracted according to the GPR-determined location, and the detected object can be interpreted as a rebar or a plastic pipe by the EMI amplitude. The GPR-estimated cover thickness range is used as a constraint for further estimation of the rebar diameter and cover thickness by calculating the mean square errors between the measured and calibrated EMI data. A laboratory experiment demonstrates that integrating GPR and EMI data can greatly enhance the estimation accuracy. The field experiments on two concrete columns show that both the rebar diameter and cover thickness can be accurately estimated. We conclude that the developed EMI-GPR dual sensor can have a promising prospect in the practical NDT of concrete structures. The method of integrating GPR and EMI data can also be used for estimation of the diameter and buried depth of other cylindrical conductive objects, such as a metal pipe.

One of the limitations of the developed system and algorithm is that it is still difficult to implement an effective measurement and estimation of a rebars in a densely-meshed rebar net, where the GPR and EMI signals from the neighboring rebars severely interfere with each other. An attempt will be made to use advanced signal processing algorithms, and another attempt will aim to improve the system performance and enhance the directivity of the sensors. It is worth noting that the nominal frequency (corresponding to the bandwidth) of the pulse GPR has a significant impact on its resolution. The larger the bandwidth of the transmitting pulse is, the thinner the hyperbolic trajectory is, resulting in a more precise hyperbola extraction with Sobel operator as well as a more accurate cover thickness estimation in the following step. Thus, we are currently attempting to develop a prototype with a higher GPR center frequency of 2.6 GHz. In addition, the data processing is time- and labor-consuming with the increasing demands of field tests. We think artificial intelligent algorithms, such as deep learning, may have the potential in improving the efficiency of data analysis and processing.

## Figures and Tables

**Table 1:** True and estimated rebar diameters and cover thicknesses in the laboratory experiment, and their relative errors.

No.	Cover Thickness				Diameter		
	True	GPR Pre-Estimated	Estimated	Error	True	Estimated	Error
#1	15 mm	11–19 mm	14 mm	6.7%	6 mm	6 mm	0
#2	21 mm	18–28 mm	21 mm	0	8 mm	8 mm	0
#3	25 mm	20–31 mm	25 mm	0	10 mm	10 mm	0
#4	37 mm	31–42 mm	36 mm	2.7%	12 mm	12 mm	0
#5	35 mm	32–43 mm	35 mm	0	14 mm	14 mm	0
#6	42 mm	38–49 mm	41 mm	2.4%	16 mm	16 mm	0
#7	32 mm	31–41 mm	33 mm	3.1%	18 mm	18 mm	0
#8	38 mm	36–47 mm	39 mm	2.6%	20 mm	20 mm	0
#9	43 mm	40–53 mm	43 mm	0	22 mm	20 mm	9.1%
#10	46 mm	43–56 mm	47 mm	2.2%	25 mm	25 mm	0
#11	50 mm	47–60 mm	50 mm	0	28 mm	28 mm	0

**Table 2:** Estimated and measured rebar diameters and cover thicknesses and their relative errors in the field test.

No.	Cover Thickness			Rebar Diameter		
	Measured	Estimated	Error	Measured	Estimated	Error
①	36.8 mm	35 mm	4.9%	24.7 mm	25 mm	1.2%
②	23.4 mm	24 mm	2.6%	27.7 mm	28 mm	1.1%
③	46.2 mm	46 mm	0.4%	24.3 mm	25 mm	2.9%
④	46.8 mm	46 mm	1.7%	24.4 mm	25 mm	2.5%

## References

[1] McCann D.M., Forde M.C. (2001). Review of NDT methods in the assessment of concrete and masonry structures. NDT E Int..

[2] Utsi V., Utsi E. Measurement of reinforcement bar depths and diameters in concrete. Proceedings of the Tenth International Conference on Grounds Penetrating Radar.

[3] Rens K.L., Wipf T.J., Klaiber F.W. (1997). Review of Nondestructive Evaluation Techniques of Civil Infrastructure. J. Perform. Constr. Facil..

[4] Gaydecki P.A., Burdekin F.M. (1994). An inductive scanning system for two-dimensional imaging of reinforcing components in concrete structures. Meas. Sci. Technol..

[5] Gaydecki P., Silva I., Fernandes B.T., Yu Z.Z. (2000). A portable inductive scanning system for imaging steel-reinforcing bars embedded within concrete. Sens. Actuators A Phys..

[6] Allidred J., Chua J., Chamberlain D. Determination of reinforcing bar diameter and cover by analysing traverse profiles from a cover meter. Proceedings of the International Symposium Non-Destructive Testing in Civil Engineering.

[7] Fernandes B.T., Silva I., Gaydecki P.A. (2000). Vector extraction from digital images of steel bars produced by an inductive scanning system using a differential gradient method combined with a modified Hough transform. NDT E Int..

[8] Quek S., Gaydecki P., Zaid M.A.M., Miller G., Fernandes B. (2003). Three-dimensional image rendering of steel reinforcing bars using curvilinear models applied to orthogonal line scans taken by an inductive sensor. NDT E Int..

[9] Sivasubramanian K., Jaya K.P., Neelemegam M. (2013). Covermeter for identifying cover depth and rebar diameter in high strength concrete. Int. J. Civ. Struct. Eng..

[10] Alldred J. Improvement to the orthogonal method for determining reinforcing bar diameter using a cover meter. Proceedings of the Sixth International Conference on Structural Faults and Repair.

[11] Zaid M., Gaydecki P., Quek S., Miller G., Fernandes B. (2004). Extracting dimensional information from steel reinforcing bars in concrete using neural networks trained on data from an inductive sensor. NDT E Int..

[12] Algernon D., Hiltunen D.R., Ferraro C.C., Ishee C. (2011). Rebar detection with cover meter and ultrasonic pulse echo combined with automated scanning system. J. Transp. Res. Board..

[13] Prego F.J., Solla M., Puente I., Arias P. (2017). Efficient GPR data acquisition to detect underground pipes. NDT E Int..

[14] Liu H., Deng Z., Han F., Xia Y., Liu Q.H., Sato M. (2017). Time-frequency analysis of air-coupled GPR data for identification of delamination between pavement layers. Constr. Build. Mater..

[15] Liu H., Sato M. (2014). In situ measurement of pavement thickness and dielectric permittivity by GPR using an antenna array. NDT E Int..

[16] Liu H., Takahashi K., Sato M. (2014). Measurement of dielectric permittivity and thickness of snow and ice on a brackish lagoon using GPR. IEEE J. Sel. Top. Appl. Earth Obs. Remote Sens..

[17] Zhou F., Miorali M., Slob E., Hu X. (2018). Reservoir monitoring using borehole radars to improve oil recovery: Suggestions from 3D electromagnetic and fluid modeling. Goephyiscs.

[18] Ciarletti V., Corbel C., Plettemeier D., Cais P., Clifford S.M., Hamran S.E. (2011). WISDOM GPR designed for shallow and high-resolution sounding of the martian subsurface. Proc. IEEE.

[19] He X., Zhu Z., Liu Q., Lu G. Review of GPR rebar detection. Proceedings of the Progress in Electromagnetics Research Symposium.

[20] Chang C.W., Lin C.H., Lien H.S. (2009). Measurement radius of reinforcing steel bar in concrete using digital image GPR. Constr. Build. Mater..

[21] Zanzi L., Arosio D. (2013). Sensitivity and accuracy in rebar diameter measurements from dual-polarized GPR data. Constr. Build. Mater..

[22] Wiwatrojanagul P., Sahamitmongkol R., Tangtermsirikul S., Khamsemanan N. (2017). A new method to determine locations of rebars and estimate cover thickness of RC structures using GPR data. Constr. Build. Mater..

[23] Kalogeropoulos A., van der Kruk J., Hugenschmidt J., Busch S., Merz K. (2011). Chlorides and moisture assessment in concrete by GPR full waveform inversion. Near Surf. Geophys..

[24] Chen W., Shen P., Shui Z. (2012). Determination of water content in fresh concrete mix based on relative dielectric constant measurement. Constr. Build. Mater..

[25] Hong S., Lai W.L., Helmerich R. (2015). Experimental monitoring of chloride-induced reinforcement corrosion and chloride contamination in concrete with ground-penetrating radar. Struct. Infrastruct. Eng..

[26] Hong S., Wiggenhauser H., Helmerich R., Dong B., Dong P., Xing F. (2017). Long-term monitoring of reinforcement corrosion in concrete using ground penetrating radar. Corros. Sci..

[27] Gu P., Beaudoin J.J. (1997). Dielectric behaviour of hardened cementitious materials. Adv. Cem. Res..

[28] AL-Qadi I.L., Lahouar S. (2005). Measuring layer thicknesses with GPR–Theory to practice. Constr. Build. Mater..

[29] Shihab S., Al-Nuaimy W. (2005). Radius estimation for cylindrical objects detected by ground penetrating radar. Subsurf. Sens. Technol. Appl..

[30] Ristic A.V., Petrovacki D., Govedarica M. (2009). A new method to simultaneously estimate the radius of a cylindrical object and the wave propagation velocity from GPR data. Comput. Geosci..

[31] Brunzell H. (1999). Detection of shallowly buried objects using impulse radar. IEEE Trans. Geosci. Remote Sens..

[32] Mechbal Z., Khamlichi A. (2017). Determination of concrete rebars characteristics by enhanced post-processing of GPR scan raw data. NDT E Int..

[33] Windsor C.G., Capineri L., Falorni P. The estimation of buried pipe diameters by generalized Hough transform of radar data. Proceedings of the Progress in Electromagnetic Research Symposium.

[34] Liu H., Xing B., Zhu J., Zhou B., Wang F., Xie X., Liu Q.H. (2018). Quantitative stability analysis of ground penetrating radar systems. IEEE Geosci. Remote Sens. Lett..

[35] Feng X., Sato M., Liu C. (2012). Subsurface imaging using a handheld GPR MD system. IEEE Geosci. Remote Sens. Lett..

[36] Van Meirvenne M., Van De Vijver E., Vandenhaute L., Seuntjens P. Investigating soil pollution with the aid of EMI and GPR measurements. Proceedings of the 15th International Conference on Ground Penetrating Radar.

[37] Yoder R.E., Freeland R.S., Ammons J.T., Leonard L.L. (2001). Mapping agricultural fields with GPR and EMI to identify offsite movement of agrochemicals. J. Appl. Geophys..

[38] Inman D.J., Freeland R.S., Ammons J.T., Yoder R.E. (2002). Soil investigations using electromagnetic induction and ground-penetrating radar in southwest Tennessee. Soil Sci. Soc. Am. J..

[39] Saey T., Delefortrie S., Verdonck L., De Smedt P., Van Meirvenne M. (2014). Integrating EMI and GPR data to enhance the three-dimensional reconstruction of a circular ditch system. J. Appl. Geophys..

[40] Gao Y., Ye S., Zhang X., Fang G. (2015). Novel detection system based on EMI and UWB radar. Electron. Meas. Technol..

[41] China National Standards GB-T 1499.2-2018 (2018). Steel for the Reinforcement of Concrete—Part 2: Hot Rolled Ribbed Bars.

[42] Cassidy N.J., Jol H.M. (2009). Ground penetrating radar data processing, modelling and analysis. Ground Penetrating Radar: Theory and Applications.

[43] Ansari M.D., Mishra A.R., Ansari F.T. (2018). New divergence and entropy measures for intuitionistic fuzzy sets on edge detection. Int. J. Fuzzy Syst..

[44] Senin S.F., Hamid R. (2016). Ground penetrating radar wave attenuation models for estimation of moisture and chloride content in concrete slab. Constr. Build. Mater..

[45] Cassidy N.J., Jol H.M. (2009). Electrical and magnetic properties of rocks, soils and fluids. Ground Penetrating Radar: Theory and Applications.

[46] Comite D., Galli A., Catapano I., Soldovieri F. (2017). The role of the antenna radiation pattern in the performance of a microwave tomographic approach for GPR imaging. IEEE J. Sel. Top. Appl. Earth Obs. Remote Sens..

[47] Pettinelli E., Di Matteo A., Mattei E., Crocco L., Soldovieri F., Redman J.D., Annan A.P. (2009). GPR response from buried pipes: Measurement on field site and tomographic reconstructions. IEEE Trans. Geosci. Remote Sens..

